# Current Role and Potential of Triple Quadrupole Mass Spectrometry in Biomedical Research and Clinical Applications

**DOI:** 10.3390/molecules29235808

**Published:** 2024-12-09

**Authors:** Andreas Tsakalof, Alexey A. Sysoev, Kira V. Vyatkina, Alexander A. Eganov, Nikolay N. Eroshchenko, Alexey N. Kiryushin, Alexey Yu. Adamov, Elena Yu. Danilova, Alexander E. Nosyrev

**Affiliations:** 1 Laboratory of Biochemistry, School of Medicine, University of Thessaly, Biopolis, 41111 Larissa, Greece; 2 Laboratory of Applied Ion Physics and Mass Spectrometry, National Research Nuclear University MEPhI (Moscow Engineering Physics Institute), 115409 Moscow, Russia; aasysoyev@mephi.ru (A.A.S.); ayadamov@mephi.ru (A.Y.A.); 3 Biomedical Science and Technology Park, I.M. Sechenov First Moscow State Medical University, 119991 Moscow, Russia; vyatkina_k_v@staff.sechenov.ru (K.V.V.); eganov_a_a@staff.sechenov.ru (A.A.E.); eroshchenko_n_n@staff.sechenov.ru (N.N.E.); kiryushin_a_n@staff.sechenov.ru (A.N.K.); danilova_e_yu_1@staff.sechenov.ru (E.Y.D.); 4 Institute of Translational Biomedicine, Saint Petersburg State University, 199034 St. Petersburg, Russia; 5 Department of Software Engineering and Computer Applications, Saint Petersburg Electrotechnical University “LETI”, 197376 St. Petersburg, Russia; 6 Department of Analytic Chemistry, Faculty of Chemistry, Lomonosov Moscow State University, 119991 Moscow, Russia

**Keywords:** mass spectrometry, clinical applications, triple quadrupole, biomedical research, tandem mass spectrometry

## Abstract

Mass-spectrometry-based assays nowadays play an essential role in biomedical research and clinical applications. There are different types of commercial mass spectrometers on the market today, and triple quadrupole (QqQ) is one of the time-honored systems. Here, we overview the main areas of QqQ applications in biomedicine and assess the current level, evolution, and trends in the use of QqQ in these areas. Relevant data were extracted from the Scopus database using the specified terms and Boolean operators defined for each field of the QqQ application. We also discuss the recent advances in QqQ and QqQ-based analytical platforms, which promote the clinical application of these systems, and explain the indicated substantial increase in triple quadrupole use in biomedicine. The number of biomedical studies utilizing QqQ increased 2–3 times this decade. Triple quadrupole is most intensively used in the field of endocrine research and testing. On the contrary, the relative rate of immunoassay utilization—a major competitor of chromatography–mass spectrometry—decreased in this area as well as its use within Therapeutic drug monitoring (TDM) and forensic toxicology. Nowadays, the applications of high-resolution accurate mass (HRAM) mass spectrometers in the investigated areas represent only a small fraction of the total amount of research using mass spectrometry; however, their application substantially increased during the last decade in the untargeted search for new biomarkers.

## 1. Introduction

**Figure 1:**
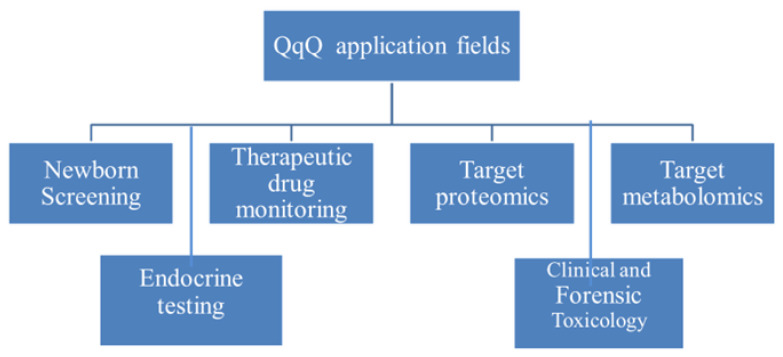
Main application areas of triple quadrupole in biomedicine.

Modern chromatography–mass spectrometry has made a significant contribution to the currently observed progress in bio-medical research and applications; in particular, it has brought a revolution in mass-spectrometry-based disease diagnosis, preventive screening, early disease detection, and the development of personalized/precision medicine [1,2,3,4,5]. Moreover, chromatography–mass spectrometry is the main instrumental platform for the emerging “omics” technologies (proteomics, metabolomics, lipidomics, etc.), which are nowadays reaching the initial translational phase from basic research to routine clinical applications. There are various types of commercial mass spectrometers on the market today, and selecting an optimal instrument for a particular task is rarely a straightforward consideration. The selection criteria can differ significantly depending on whether the instrument is intended for routine measurements or basic research. In this review, we focus on the current role and potential of the triple quadrupole mass analyzer. The first triple quadrupole mass spectrometer (QqQ) was presented by Christie G. Enke and Richard A. Yost in the late 1970s [6], and our review outlines that it is nowadays the main mass spectrometer used in biomedical research and clinical applications. There is little doubt that this type of instrument will hold this position in the future due to its relatively low cost and variety of modes of operation that make it possible to develop methods with superior specificity and sensitivity. Triple quadrupole mass spectrometers are produced by leading manufacturers of mass spectrometers who are constantly working on improving the features and merits of these devices (Supplementary Table S1). Nowadays, QqQ instruments are used in various application fields, as outlined in Figure 1; however, the extent of their use in each of the identified areas varies. In this review, we aimed to assess the current state of the art and demonstrate the evolution and trends of QqQ use in these areas. We also discussed recent advances in chromatography–QqQ that reflect these trends and are driving wider adoption of this analytical platform in biomedical research and clinical laboratories.

## 2. Methods of Data Acquisition

Relevant data were extracted from the Scopus database using specified terms and Boolean operators defined for each field and indicated in the corresponding section. The search was limited to “articles” and excluded other document types, e.g., reviews, book chapters, conference papers, and editorials. The specified terms were searched for in the article’s title, abstract, and keywords. The triple quadrupole mass analyzer is also often referred to as tandem mass spectrometry or MS/MS, and these terms were united by the Boolean operator “OR”. To assess the dynamics of the QqQ utilization rates, the search was conducted independently for two decades: 2004–2014 and 2014–2024. The applications of other tandem configurations, such as Q-TOF and hybrid Orbitrap, were also searched for separately to evaluate their impact on the investigated areas. The extent of use of immunoassay, a major competitor of chromatography–mass spectrometry, was also evaluated.

## 3. Results and Discussion: Current Status and Trends in the Use of Triple Quadrupole in Clinical Applications and Biomedical Research

### 3.1. Newborn Screening

Newborn screening (NBS) involves a set of clinical tests of biological samples from newborns for the early, pre-symptomatic detection of congenital abnormalities critical to the child’s health. Early diagnosis makes it possible to take preventive measures to preserve child health and reduce child mortality and morbidity [7,8]. Conclusions are drawn based on specific metabolic biomarkers of these abnormalities [8,9]. All developed countries and many developing countries have implemented NBS programs for mandatory testing of infants for a number of congenital diseases, and this number is steadily increasing [10,11]. Thus, starting in 2023, a program of expanded newborn screening is included in the commitments of neonatal services in the Russian Federation, and the testing of newborns has grown from 5 to 36 congenital diseases [12]. The feasibility of such expansion can be largely attributed to the introduction of LC-MS/MS to NBS laboratories in the 1990s [13]. Nowadays, liquid chromatography coupled to a triple quadrupole mass analyzer is the main instrumental platform for routine measurements and research in NBS programs. Thus, a Scopus search using the terms and Boolean operators indicated in Table 1 reveals that in the last decade (2014–2024) at least 84% (924 out of 1098) of studies involved in newborn screening utilized mass spectrometry in their research. In particular, 823 out of those 924 papers explicitly mention tandem mass spectrometry or triple quadrupole. Only a few studies (seven) reported the use of other tandem configurations such as Q-TOF or hybrid Orbitrap MS. This preference for the triple quadrupole mass analyzer is due to the relatively low price of these instruments and, at the same time, their superior specificity and sensitivity in target analysis achieved by two consecutive mass filtering stages, (Section 4.2) despite the low resolution of the single quadrupole mass analyzer. The number of studies in newborn/neonatal screening significantly increased compared to the previous decade (2004–2014, Table 1). Immunoassays, the main alternative to the mass spectrometry approach to quantify different compounds, were also used at the rate of about 15–17% of all the studies in the previous two decades (Table 1).

### 3.2. Endocrine Testing

Endocrine testing focuses on detecting endocrine disorders and relies significantly on measuring hormone levels in biological samples. Endocrine disorders comprise a large list of diseases (>30) that have a negative impact on human well-being as well as a considerable economic burden on society [14]. However, if detected, most of these disorders are treatable and some are easily treated, e.g., thyroid dysfunctions. Accurate quantification of hormones is crucial for the proper diagnosis of disease, and recognizing the importance of these measurements, the 1977 Nobel Prize in Physiology or Medicine was awarded to Rosalyn C. Yalow for the development of the radioimmunoassay (RIA) for precise and specific measurements of the concentrations of peptide hormones and many other biologically significant substances in blood and other body fluids [15]. In the late 1980s, radioactive labels were replaced with chemiluminescent labels and, nowadays, direct chemiluminescent immunoassays (CLIA) are the most frequently used immunoassays for the measurement of hormones [16]. However, immunoassays, along with their advantages, have several significant disadvantages (Section 4), among which the most significant is the inadequate specificity of these tests due to the cross-reactivity of antibodies [17]. This can lead to a large number of false positives, irreproducible results, and overestimated concentrations. On the contrary, mass spectrometry offers superior specificity and more accurate and reliable results. Moreover, in contrast to immunoassays, MS is a multi-component method that allows for multiple steroid detection and steroid profiling for the complex evaluation of steroidogenesis in the organism [18,19]. Given these advantages, endocrine testing shifts strongly nowadays to mass-spectrometry-based assays [20]. Thus, according to the United Kingdom National External Quality Assessment Service (UK NEQAS) steroid hormone proficiency testing scheme, the number of participants using LC-MS/MS increased from 3% to 18% from 2011 to 2019 [20]. This trend was also clearly demonstrated by the recommendation from the editors of the *Journal of Clinical Endocrinology and Metabolism* to avoid using immunoassays and instead use MS for the measurement of sex steroids [21]. The LC–tandem MS (triple quadrupole) is also identified as the reference procedure measurement (RPM) in Clinical Standardization Programs organized by the Centers for Disease Control and Prevention (CDC) [22]. A reference procedure (also known as a reference method) is a measurement method that has been officially defined as the gold standard for a specific analysis. It demonstrates appropriate trueness (accuracy) and precision (reproducibility) for its intended use. The critical advantage of mass-spectrometry-based methods is the ability to profile steroids by simultaneous multiple steroid quantification in a single analysis. Thus, entire metabolic pathways of steroidogenesis can be investigated, and the coverage of endocrine diseases can be expanded in the field [23]. A trend can be observed for developing new methods that cover the increased part of the whole steroidome—a comprehensive collection of steroids present in a biological system [24]. For example, a method based on GC–triple quadrupole MS for the determination of a hundred endogenous steroids in human serum was recently presented [25]. Endocrine research is the most intensive and intensively developing area of application of hybrid mass spectrometry, represented by a large number of publications that have more than doubled over the previous decade (14,968 vs. 6625 studies, Table 2). Mass spectrometry is now used in most endocrine studies, but immunoassays are also popular, apparently because of their ease of implementation; however, the proportion of immunoassays has declined from about 36% in the previous decade to 29% in the current decade. It can be concluded from the data retrieved (Table 2) that the extent of QqQ use (mostly referred to as tandem mass spectrometry) in endocrine research nowadays is notable and has also significantly increased compared to the previous decade. The prevalence of QqQ use is obviously much higher than indicated in Table 2 due to the following reasons: First, in many publications, authors do not specify the type of mass analyzer used in search sections such as the article’s title, abstract, and keywords. Second, only a few studies focusing on endocrine research used high-resolution Q-TOF and Orbitrap (Table 2).

### 3.3. Therapeutic Drug Monitoring (TDM)

Therapeutic drug monitoring (TDM) specializes in measuring circulating drug levels and is an important component of the concept of personalized/precision medicine [26]. Due to human genetic polymorphism, drug–drug interactions (when multiple drugs are administered), and drug–disease interactions, high variability in the pharmacokinetics of drugs may be observed and, therefore, the dosing regimen of drugs should be personalized for the most effective treatment and/or prevention of drugs’ toxic effects. This is particularly important for drugs with a low therapeutic index (small difference between the minimum effective concentration and the minimum toxic concentration in the blood), for example, a variety of antiviral drugs, antibiotics, antiepileptics, psychotropics, and immunosuppressive agents [27,28]. Currently, mass-spectrometry-based methods dominate in TDM, and their applications have increased significantly over the last ten years (Table 3). During the last decade, 2014–2024, about 1492 studies used mass spectrometry for TDM, of which 1235 indicated the use of triple quadrupole, referred to as triple quadrupole or tandem mass spectrometry. Only a few studies used Q-TOF or Orbitrap MS. Thus, it can be concluded from the retrieved data that triple quadrupole is the main analyzer used nowadays for TDM as only a few applications of another tandem MS, viz., Q-TOF or Orbitrap, were retrieved (Table 3).

This area of mass spectrometry application will expand as the incorporation of precision medicine into clinical practice has been identified as a priority of national and international health policy [29,30]. The European Commission recognizes the importance of personalized medicine and has established the European Partnership for Personalized Medicine. This partnership aims to promote all areas and disciplines of precision medicine, facilitate innovation, and integrate it into health systems for continuous improvement [31,32].

### 3.4. Clinical and Forensic Toxicology

Clinical and forensic toxicology are two main fields of analytical toxicology that use common analytical techniques to detect, identify, and quantify xenobiotics that cause poisoning or fatal intoxication. In the case of clinical toxicology, the acquired data assist in the diagnosis and treatment of poisoning, while in the case of forensic toxicology, the results assist in the medical–legal investigation of death (postmortem forensic toxicology), abnormal human behavior (human performance forensic toxicology), or use of illicit substances (forensic drug testing). Forensic toxicology data are used as evidence in legal proceedings and special demands are made on their quality. Therefore, during the last two decades, mass spectrometry has replaced less specific identification techniques in forensic toxicology, and strict criteria for accepting mass spectrometry data and quality control in Forensic Toxicology Laboratories have been established [33]. Chromatography–mass spectrometry has a high identification power in conformity with the “identification point” system that has been developed for rating and comparing different identification techniques [34]. Non-mass-spectrometric techniques, e.g., colorimetric tests, immunoassays, and chromatography coupled with non-MS detectors (NPD, ECD, and fluorescence), have low identification power and cannot achieve point scores—defined as the lowest score for unambiguous compound identification. They can be used for screening and should be confirmed by a verification method [34,35]. The verification method enables the unequivocal identification and, if necessary, quantification of the compound. Hyphenated mass spectrometry is a method of choice for confirmation. Until recently, xenobiotic detection and identification workflow comprised rapid screening by immunoassay and subsequent confirmation of positive results by LC- or GC-MS [36]; however, the recent significant simplification and acceleration of chromatography–mass spectrometry analysis (see Section 6) allows for screening and identification to be performed solely by this method, which has sufficient identification power. The identification point system drives laboratories to use chromatography–mass spectrometry as the most powerful instrumentation platform for the identification of xenobiotics. The number of publications in the field of forensic toxicology performed with the use of mass spectrometry techniques doubled in comparison with the previous decade (Table 4). The number of studies that directly indicated the use of the triple quadrupole also doubled. Only a relatively small percentage of published studies indicated the use of HRAM Q-TOF and Orbitrap (Table 4). However, the application of HRAM mass spectrometry will increase for the detection and identification of newly synthesized illicit New Psychoactive Substances (NPS) [37]. These substances are introduced into the market yearly in large diversity and quantities [38], and targeted analytical approaches are unsuitable for their detection and identification.

Unlike forensic toxicology, in clinical toxicology, the turnaround time (TAT, or results waiting time) is an important criterion for the method’s clinical utility for diagnosis and treatment of poisoning [39]. In particular, for an antidote administration, the poison must be identified shortly after the patient is admitted to the hospital. Thus, in case of acetaminophen poisoning, the administration of antidote N-acetylcysteine is effective within 8–12 h of drug ingestion. In the case of acetaminophen and some other toxins, the treatment depends on their concentration in the biological sample [40,41]. Rapid point-of-care (POC) or laboratory tests are most suitable to offer the desired information, and nowadays, immunoassays and chemical tests are dominant methods due to the rapid TAT [42]. In combination with observed patient symptoms, the patient’s history, and circumstantial evidence (e.g., empty drug containers), these tests provide sufficient information to guide the patient’s treatment. Immunoassays continue to evolve, providing enhanced sensitivity and multiplexing capabilities for the rapid detection of multiple drugs in a single test and they will obviously continue to be applied in emergency toxicology. Hyphenated MS assays have a slow turnaround time and, thus, for the time being, cannot contribute significantly to decisions for patient treatment in the emergency department. This can explain the relatively small number of publications retrieved with the terms {clinical toxicology} and {mass spectrometry} (Table 5). However, the analytical advantages of mass spectrometry have stimulated the development of new MS-based technologies to meet the main requirements of clinical toxicology—rapid TAT, high sample throughput, and availability at the point of care. An example of a system with high throughput and analysis speed is the RapidFire365-MS/MS online solid-phase extraction mass spectrometry system from Agilent Technologies Inc. [43]. In this technology, the ultra-fast RapidFire 365 automated solid-phase extraction system is directly integrated with the triple quadrupole mass spectrometer without the use of LC. With RapidFire365-MS/MS, the sample analysis time is usually <10–15 s. With the use of this technology, 18,000 urine samples were screened for 14 synthetic cannabinoids and synthetic cathinones within 4 weeks with a total instrument time of 55 h [44]. These samples were previously analyzed by traditional LC-MS/MS within 3 months and the total instrument time was 2850 h.

### 3.5. Targeted Metabolomics and Proteomics

**Figure 2:**
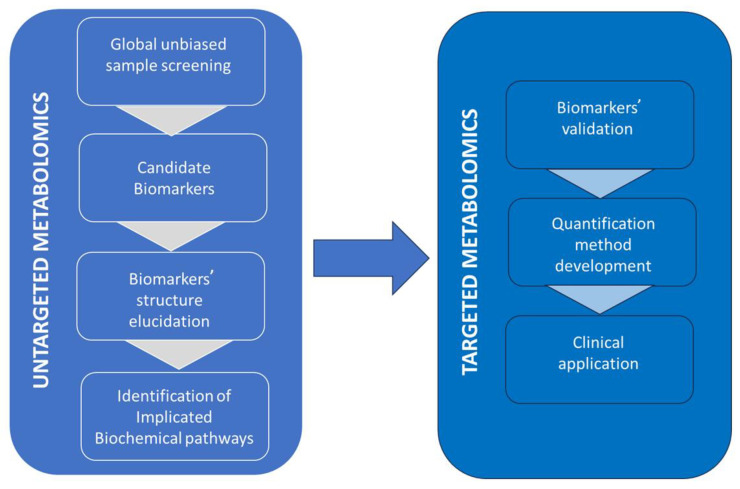
Complimentary role of untargeted and targeted metabolomics in biomedical research. Biomarkers (e.g., of disease) are discovered by untargeted metabolomics and then implicated in clinical practice with the use of targeted metabolomics.

Metabolomics is a relatively new field of research that evaluates the performance of various biochemical reactions/processes in living organisms by tracking the metabolites and the end or intermediate products of these reactions. Alterations in metabolites’ levels or profiles testify to abnormalities in the relevant biochemical processes caused by pathophysiologic reasons. Identification of these alterations has proved to be a useful tool clinically for disease screening and diagnosis as well as for the monitoring of therapeutic outcomes [45]. Metabolomics is also increasingly used for clarifying disease biochemical backgrounds (pathophysiology) and using this knowledge for the definition of optimal drug targets and the development of new drugs [46]. While genomics gives information about what can happen in the organism, metabolomics indicates what is currently happening, the metabolic phenotype of the organism. Nowadays, two approaches are implemented in metabolomics: untargeted and targeted. Target metabolomics measures predetermined target metabolites and compares them to established reference levels for clinical decision-making. In contrast, untargeted metabolomics aims to discover new metabolite biomarkers of disease by analyzing in an unbiased screening mode all ions produced in the ion source and detecting chromatographic peaks without information about the corresponding compound’s nature. By multi-step data treatment and analysis from compared population groups, e.g., patients and controls, putative metabolites-biomarkers are identified and, subsequently, their structures are elucidated (Figure 2) [47,48]. However, processing the large amounts of data acquired is time-consuming and does not fit the demand for quick analysis and high throughput in clinical laboratories. Thus, nowadays, these two approaches have complementary roles with untargeted metabolomics used for biomarker discovery and targeted metabolomics for their implication in clinical practice within clinical laboratories (Figure 2). High-resolution accurate mass (HRAM) mass spectrometers offer more powerful capabilities for unveiling unknown compounds’ structures. Orbitrap and TOF-based systems are the most widely used HRAM mass spectrometers for untargeted metabolomics, while triple quadrupole-based systems, GC-MS/MS, or LC-MS/MS are the most popular for targeted metabolomics.

The term “metabolome” was first introduced in 1998 [49]; however, GC-MS protocols for investigating key metabolic pathways such as the tricarboxylic acid (TCA) cycle, glycolysis, and the pentose phosphate pathway were developed substantially earlier [50,51]. Our search retrieved rather few (75) research articles on targeted metabolomics with the use of mass spectrometry for the 2004–2014 decade (Table 6). This research was tremendously intensified during the last decade, 2014–2024, with 1177 publications on the same topic. From these publications, 947 clarified that tandem mass spectrometry was used.

Another relatively new field of triple quadrupole MS application is expression proteomics, which studies the quantitative and qualitative expression of proteins [52]. The concept of the proteome was introduced in 1995 and characterizes the “total protein complement able to be encoded by a given genome” [53]. Today, the study of the proteome (proteomics) plays an important role in biomedicine in the fields of disease diagnosis and drug research and development. The use of triple quadrupole MS in MRM mode is very popular for the quantification of targeted proteins and identification of proteins’ post-translational modifications [54]. Targeted MRM-MS proteomics is a reproducible, accurate, and multiplex method for quantifying protein biomarkers that provide information on the biological status of the organism. The high multiplexing power of QqQ MS is one of the main advantages over enzyme-linked immunosorbent assays (ELISAs) that have been the dominant technique for measuring the absolute abundance of protein in complex mixtures but only one protein at a time [55]. Thus, for example, 136 urinary proteins were precisely quantified by LC/MRM-MS on a triple quadrupole mass spectrometer [56]. Also of note is the wide range of concentrations of quantified proteins from 8.6 µg/mL to 25 pg/mL (over five orders of magnitude). The triple quadrupole MS dominates in the field of targeted proteomics and its application has tripled in comparison with the previous decade (Table 7).

## 4. Recent Advances in Triple Quadrupole Mass Analyzer Technology and QqQ-Based Methodologies

From the above analysis, we can conclude that immunoassays remain the main competitors of QqQ-based methods. The former has several advantages (Table 8), which at the same time are the main disadvantages when implementing MS-based methods in biomedical applications. However, in recent years, new developments have largely eliminated these disadvantages and facilitated the application of MS-based methods. These developments make chromatography–mass spectrometry more accessible and increase the speed of analysis.

### 4.1. Making the Chromatography–Mass Spectrometry More Accessible—Automatization of Method Development and Application

Developing the LC-MS or GC-MS method is a complex and time-consuming process requiring an analyst with a high level of knowledge and experience. Automatization of the method’s development, optimization, and measurement quality control/quality assurance intend to address the drawbacks of the challenging method development and implementation and the need for specialized staff. Software for automatic chromatographic method development and optimization has been created and commercialized [57,58,59]. Software to help automate the development of targeted triple quadrupole methods has also been introduced to the market [60]. This software can optimize MRM’s specific parameters and ion source parameters in a fully automated or semi-automated fashion [60]. Another important feature is the automatic system troubleshooting, which provides timely notification of maintenance needs. All these software developments simplify the use of LC-MS and GC-MS instrumentation and make their application in biomedical research more attractive. Method development can be significantly simplified using LC-MS/MS complete kits supplied by specialized providers (e.g., Chromsystems™ (Gräfelfing, Germany), Recipe™ (Vaughan, ON, USA), and Waters™ (Milford, MA, USA)). These kits include all the reagents and materials to transfer the method developed by the provider to the laboratory applying the method: analytical columns, mobile phases, controls, and calibrators. LC-MS/MS kits for TDM, newborn screening, steroids, and amino acid analyses are available on the market.

### 4.2. Increasing the Throughput Rate by Reducing the Analysis Time

**Figure 3:**
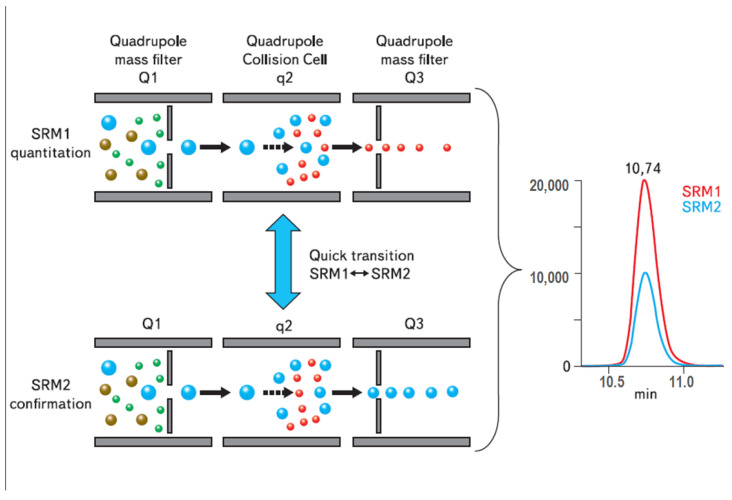
Operation of triple quadrupole MS in Multiple Reaction Monitoring (MRM) mode. Q1, Q2, and Q3 are constituent parts of triple quadrupole MS. In MRM mode Q1 and Q3 represent two mass filters for precursor and product ion selection, while Q2 (collision cell) creates fragment ions via collisionally induced dissociation (CID). The first mass filter Q1 selects the desired precursor ion with defined *m*/z* from the ions produced by the compound fragmentation in the ion so*urce. However, due to the low resolution of a quadrupole mass filter, the ions within *m*/*z* ± 1 from other co-eluting compounds/contaminants can also pass Q1 filter (where ± 1 is the typical resolution of quadrupole mass filter, nowadays much improved (Supplementary Table S1)). After passing Q1, the ions are fragmented in Q2. Q3 selects only product ions specific to the desired precursor ion. There is very little probability that other ions within *m*/*z* ± 1 have the same product ions and these two levels of mass selection ensure high selectivity of QqQ. Due to high selectivity, background noise is minimized, resulting in a higher signal-to-noise ratio and increased sensitivity. The transition from precursor ion to product ion is a fragmentation reaction. Morden QqQ mass spectrometer can monitor about 500 fragmentation reactions per second (Supplementary Table S1). Usually, two to four transitions are monitored using one product ion for quantitation (quantifier or target ion) and the other product ions (qualifier ion/ions) for confirmation of the analyte’s identity. The ratio of the intensities of the quantifier and qualifier ions must be within the tolerance limits.

**Figure 4:**
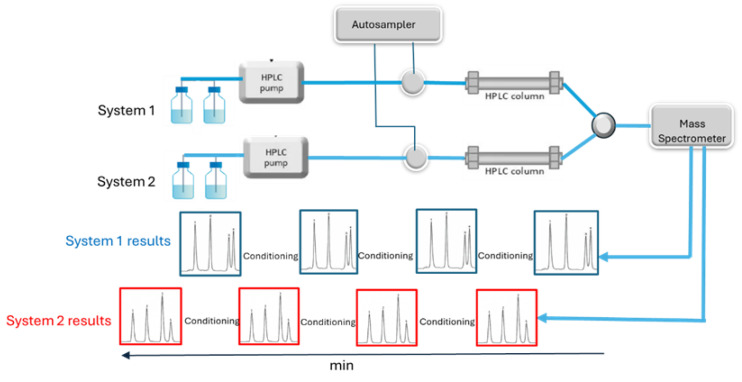
LC–multiplexing (duplexing) by connecting the two independent LC systems to one mass spectrometer in alternating mode (blue arrows). Each system is connected to the mass spectrometer in turn and only when the target components are eluting (dark-blue frames). Meanwhile, column conditioning and preparation for sample introduction are performed in the second system (red frames). Two different applications can be run in parallel (increased flexibility) or the same application on two LC systems (increased throughput).

The latest developments both in chromatography and mass spectrometry have increased the throughput rated of LC-MS and GC-MS. The shift from HPLC to UHPLC (or UPLC) started in 2004 and led to a significant increase in chromatographic resolution and a reduction in separation time [61]. To couple mass spectrometry with the UHPLC data, the acquisition rate by mass spectrometry was increased to fit the reduction in chromatographic peak width from more than 10 s (HPLC) to 1–2 s (UHPLC), and sufficient data points (at least 10) were collected to ensure an excellent chromatographic peak shape and, finally, excellent quantitation. For the full-scan mode, this was achieved by a considerable increase in scan speed from ~650 Da/s in 1970 to ~10,000 in 2012 [62] and up to 30,000 Da/s nowadays (depending on the model, Supplementary Table S1). Concerning Multiple Reaction Monitoring (MRM, Figure 3), one of the most widely used MS modes today, the implementation of algorithms for scheduling MRM according to the elution time of the components of interest significantly increased the number of transitions to be monitored concurrently (up to 500 transitions per second) and, thus, the number of analytes that can be quantified in a single run. Moreover, this scheduling removes the requirement for baseline chromatographic resolution and enables the quantification of co-eluting compounds (Figure 4). Thus, by the application of scheduled MRM, 151 pesticides were quantified by UHPLC-QqQ-MS/MS in 12 min [63]. Different vendors have developed these algorithms and have different trademarks; AB SCIEX (Framingham, MA, USA) calls this approach Scheduled MRM™, Agilent (Santa Clara, CA, USA) named it Dynamic MRM, while Thermo (Waltham, MA, USA) calls it “timed-SRM” but they all work in basically the same way.

A significant increase in sample throughput rate was also achieved in the above-mentioned RapidFire™-MS/MS system with an automated online solid-phase extraction directly integrated with the triple quadrupole MS [43]. This direct integration was made possible by advances in mass spectrometry, enabling the quantification of co-eluting compounds.

Sample preparation or sample pre-treatment has been the most time-consuming and error-prone step in sample analysis, which is mainly responsible for the low throughput of GC-MS- and LC-MS-based methods. This bottleneck was addressed by the development of automated (robotic) sample preparation systems and online with column-switching sample treatment techniques [64,65,66,67]. With the successful introduction of automation, the cost of analysis falls and the acquired data quality improves. Many instrumental solutions for automated sample preparation are currently available on the market, which are reviewed in detail in other publications [68,69].

## 5. Future Perspectives: The Transformation of LC-MS/MS to a Fully Automated Clinical Analyzer

The complete automation of the analysis by integrating most steps in one device is the key to implementing LC-MS/MS into routine clinical settings. In the last decade, such efforts were made to reduce the operator’s involvement in the process and, thus, make this high-performance technology available to less-trained staff, accelerate the analysis, and increase its throughput [70]. A milestone of these efforts is the all-in-one Cascadion™ SM Clinical Analyzer (Thermo Fisher Scientific, Vantaa, Finland) launched in 2018 [71]. This analyzer comprises a fully automated sample preparation compartment, two parallel LC channels, and triple quadrupole MS equipped with an electrospray ionization interface. Triple quadrupole MS was selected as the most suitable technology for targeted quantitative analysis, mainly implemented in clinical laboratories and referred to in the above sections. Two separate chromatographic channels with multiplexing capability (Figure 4) ensure up to 25 samples/h (serum or plasma) throughput or two different assays to run parallel into a single MS [72,73].

## 6. Conclusions

The observed progress in biomedical research and applications is directly related to the increasing use of mass spectrometry, and QqQ is the primary mass analyzer, the “workhorse”, used in the previous two decades. The number of biomedical studies utilizing QqQ has seen a 2–3-fold increase in the current decade compared to the previous. The triple quadrupole is most intensively used in the field of endocrine research and testing, it dominates in newborn screening and therapeutic drug monitoring, and is utilized in the emerging fields of target metabolomics and proteomics. Recent advances in chromatography–QqQ MS significantly reduced analysis time and increased the throughput of analysis. Automating or simplifying the crucial steps in LC-MS/MS method development and application reduced the demand for highly educated specialists to implement this technique and made it more attractive and available for routine clinical applications. Indeed, according to the data from different quality assurance programs, the number of laboratories using an LC-MS platform for routine applications increased, e.g., in endocrine testing from 3% to 18% from 2011 to 2019 and in 25-hydroxyvitamin D measurements from 0 in 2004 to 17% in 2017 and 21% in 2021 [74,75]. On, the contrary, the relative rate of immunoassay utilization—a major competitor of chromatography–mass spectrometry—decreased in endocrine research and as well as in therapeutic drug monitoring (TDM) and forensic toxicology.

Thus, taking into account the constant work of the main manufacturers to improve triple quadrupole figures of merit as well as the observed expansion of its use in biomedicine, it can be expected that triple quadrupole mass spectrometry will continue to hold a significant share in biomedical research and will be more extendedly used in routine clinical applications.

## Figures and Tables

**Table 1:** Terms, Boolean operators, and limitations (years, document type) used for the evaluation of QqQ application rate in NBS screening and research.

Terms and Boolean Operators	Retrieved Documents	
	2014–2024	2004–2014
	Number of Publications	
(TITLE-ABS-KEY ({neonatal screening}) OR TITLE-ABS-KEY ({newborn screening}) AND TITLE-ABS-KEY ({mass spectrometry})) AND (LIMIT-TO (DOCTYPE, “ar”))	924	587
(TITLE-ABS-KEY ({newborn screening}) OR TITLE-ABS-KEY ({neonatal screening}) AND TITLE-ABS-KEY ({tandem mass spectrometry}) OR TITLE-ABS-KEY ({triple quadrupole}) OR TITLE-ABS-KEY (ms/ms)) AND (LIMIT-TO (DOCTYPE, “ar”))	823	562
(TITLE-ABS-KEY ({newborn screening}) OR TITLE-ABS-KEY ({neonatal screening}) AND TITLE-ABS-KEY ({Q-TOF})) AND (LIMIT-TO (DOCTYPE, “ar”))	1	0
(TITLE-ABS-KEY ({newborn screening}) OR TITLE-ABS-KEY ({neonatal screening}) AND TITLE-ABS-KEY ({Orbitrap})) AND (LIMIT-TO (DOCTYPE, “ar”))	6	2
(TITLE-ABS-KEY ({newborn screening}) OR TITLE-ABS-KEY ({neonatal screening}) AND TITLE-ABS-KEY ({immunoassay})) AND PUBYEAR > 2003 AND PUBYEAR < 2015 AND (LIMIT-TO (DOCTYPE, “ar”))	167	92

**Table 2:** Terms, Boolean operators, and limitations (years, document type) used for the evaluation of QqQ application rate in endocrine testing and research.

Terms and Boolean Operators	Retrieved Documents	
	2014–2024	2004–2014
	Number of Publications	
((TITLE-ABS-KEY ({hormone) OR TITLE-ABS-KEY ({steroid}) OR TITLE-ABS-KEY ({endocrine}) AND TITLE-ABS-KEY ({mass spectrometry})) AND (LIMIT-TO (DOCTYPE, “ar”))	14,968	6625
(TITLE-ABS-KEY ({hormone}) OR TITLE-ABS-KEY ({steroid}) OR TITLE-ABS-KEY ({endocrine}) AND TITLE-ABS-KEY ({tandem mass spectrometry}) OR TITLE-ABS-KEY ({triple quadrupole}) OR TITLE-ABS-KEY ({ms/ms}) AND (LIMIT-TO (DOCTYPE, “ar”))	5193	2391
(TITLE-ABS-KEY ({hormone}) OR TITLE-ABS-KEY ({steroid}) OR TITLE-ABS-KEY ({endocrine}) AND TITLE-ABS-KEY (q-tof)) AND PUBYEAR > 2003 AND (LIMIT-TO (DOCTYPE, “ar”))	204	40
(TITLE-ABS-KEY ({hormone}) OR TITLE-ABS-KEY ({steroid}) OR TITLE-ABS-KEY ({endocrine}) AND TITLE-ABS-KEY (orbitrap)) AND (LIMIT-TO (DOCTYPE, “ar”))	243	48
(TITLE-ABS-KEY ({hormone}) OR TITLE-ABS-KEY ({steroid}) OR TITLE-ABS-KEY ({endocrine}) AND TITLE-ABS-KEY (immunoassay)) AND (LIMIT-TO (DOCTYPE “ar”))	6121	3746

**Table 3:** Terms, Boolean operators, and limitations (years, document type) used for the evaluation of QqQ application rate in TDM.

Terms and Boolean Operators	Retrieved Documents	
	2014–2024	2004–2014
	Number of Publications	
(TITLE-ABS-KEY ({therapeutic drug monitoring}) AND TITLE-ABS-KEY ({mass spectrometry})) AND (LIMIT-TO (DOCTYPE, “ar”))	1492	486
(TITLE-ABS-KEY ({therapeutic drug monitoring}) AND TITLE-ABS-KEY ({tandem mass spectrometry}) OR TITLE-ABS-KEY ({triple quadrupole}) OR TITLE-ABS-KEY (ms/ms)) AND (LIMIT-TO (DOCTYPE, “ar”))	1235	439
(TITLE-ABS-KEY ({therapeutic drug monitoring}) AND TITLE-ABS-KEY (q-tof)) AND (LIMIT-TO (DOCTYPE, “ar”))	3	-
(TITLE-ABS-KEY ({therapeutic drug monitoring}) AND TITLE-ABS-KEY (rbitrap)) AND (LIMIT-TO (DOCTYPE “ar”))	18	1
(TITLE-ABS-KEY ({therapeutic drug monitoring}) AND TITLE-ABS-KEY (immunoassay)) AND (LIMIT-TO (DOCTYPE, “ar”))	356	214

**Table 4:** Terms, Boolean operators, and limitations (years, document type) used to evaluate mass spectrometry and QqQ application rate in forensic toxicology.

Terms and Boolean Operators	Retrieved Documents	
	2014–2024	2004–2014
	Number of Publications	
(TITLE-ABS-KEY ({forensic toxicology}) AND TITLE-ABS-KEY ({mass spectrometry})) AND (LIMIT-TO (DOCTYPE, “ar”))	1474	860
(TITLE-ABS-KEY ({forensic toxicology}) AND TITLE-ABS-KEY ({tandem mass spectrometry}) OR TITLE-ABS-KEY ({triple quadrupole}) OR TITLE-ABS-KEY (ms/ms)) AND (LIMIT-TO (DOCTYPE, “ar”))	1268	689
(TITLE-ABS-KEY ({forensic toxicology}) AND TITLE-ABS-KEY ({orbitrap})) AND (LIMIT-TO (DOCTYPE, “ar”))	35	6
(TITLE-ABS-KEY ({forensic toxicology}) AND TITLE-ABS-KEY ({Q-TOF})) AND (LIMIT-TO (DOCTYPE, “ar”))	14	5
(TITLE-ABS-KEY ({forensic toxicology}) AND TITLE-ABS-KEY ({immunoassay})) AND (LIMIT-TO (DOCTYPE, “ar”))	86	73

**Table 5:** Terms, Boolean operators, limitations (years, document type) used for the evaluation of mass spectrometry and QqQ application rate in clinical toxicology.

Terms and Boolean Operators	Retrieved Documents	
	2014–2024	2004–2014
	Number of Publications	
(TITLE-ABS-KEY ({clinical toxicology}) AND TITLE-ABS-KEY ({mass spectrometry})) AND (LIMIT-TO (DOCTYPE, “ar”))	141	80
(TITLE-ABS-KEY ({clinical toxicology}) AND TITLE-ABS-KEY ({tandem mass spectrometry}) OR TITLE-ABS-KEY ({triple quadrupole}) AND TITLE-ABS-KEY (ms/ms)) AND (LIMIT-TO (DOCTYPE, “ar”))	112	69
(TITLE-ABS-KEY ({clinical toxicology}) AND TITLE-ABS-KEY ({orbitrap})) AND (LIMIT-TO (DOCTYPE, “ar”))	6	1
(TITLE-ABS-KEY ({clinical toxicology}) AND TITLE-ABS-KEY ({Q-TOF})) AND (LIMIT-TO (DOCTYPE, “ar”))	3	0
(TITLE-ABS-KEY ({clinical toxicology}) AND TITLE-ABS-KEY ({immunoassay})) AND (IMIT-TO (DOCTYPE, “ar”))	17	9

**Table 6:** Terms, Boolean operators, limitations (years, document type) used for the evaluation of mass spectrometry and QqQ application rate in targeted metabolomics.

Terms and Boolean Operators	Retrieved Documents	
	2014–2024	2004–2014
	Number of Publications	
(TITLE-ABS-KEY ({targeted metabolomics}) AND TITLE-ABS-KEY ({mass spectrometry}) AND NOT TITLE-ABS-KEY ({non-targeted metabolomics) OR TITLE-ABS-KEY ({untargeted metabolomics}) AND (LIMIT-TO (DOCTYPE, “ar”))	1177	75
(TITLE-ABS-KEY ({targeted metabolomics}) AND TITLE-ABS-KEY ({tandem mass spectrometry}) OR TITLE-ABS-KEY ({triple quadrupole}) OR TITLE-ABS-KEY (ms/ms) AND NOT TITLE-ABS-KEY ({non-targeted metabolomics}) AND NOT TITLE-ABS-KEY ({untargeted metabolomics})) AND (LIMIT-TO (DOCTYPE, “ar”))	947	65
(TITLE-ABS-KEY ({targeted metabolomics}) AND TITLE-ABS-KEY ({orbitrap}) AND NOT TITLE-ABS-KEY ({non-targeted metabolomics}) AND NOT TITLE-ABS-KEY ({untargeted metabolomics})) AND (LIMIT-TO (DOCTYPE, “ar”))	29	0
(TITLE-ABS-KEY ({targeted metabolomics}) AND TITLE-ABS-KEY ({Q-TOF}) AND NOT TITLE-ABS-KEY ({non-targeted metabolomics}) AND NOT TITLE-ABS-KEY ({untargeted metabolomics})) AND (LIMIT-TO (DOCTYPE, “ar”))	20	0
(TITLE-ABS-KEY ({targeted metabolomics}) AND TITLE-ABS-KEY ({immunoassay})) AND (LIMIT-TO (DOCTYPE, “ar”))	11	0

**Table 7:** Terms, Boolean operators, and limitations (years, document type) used to evaluate mass spectrometry and QqQ application rate in targeted proteomics.

Terms and Boolean Operators	Retrieved Documents	
	2014–2024	2004–2014
	Number of Publications	
(TITLE-ABS-KEY ({targeted proteomics}) AND TITLE-ABS-KEY ({mass spectrometry}) AND NOT TITLE-ABS-KEY ({non-targeted proteomics}) AND NOT TITLE-ABS-KEY ({untargeted proteomics})) AND (LIMIT-TO (DOCTYPE, “ar”))	626	205
(TITLE-ABS-KEY ({targeted proteomics}) AND TITLE-ABS-KEY ({tandem mass spectrometry}) OR TITLE-ABS-KEY ({triple quadrupole}) OR TITLE-ABS-KEY (ms/ms) AND NOT TITLE-ABS-KEY ({non-targeted proteomics}) AND NOT TITLE-ABS-KEY ({untargeted proteomics})) AND (LIMIT-TO (DOCTYPE, “ar”))	404	126
(TITLE-ABS-KEY ({targeted proteomics}) AND TITLE-ABS-KEY ({Orbitrap}) AND NOT TITLE-ABS-KEY ({non-targeted proteomics}) AND NOT TITLE-ABS-KEY ({untargeted proteomics})) AND (LIMIT-TO (DOCTYPE, “ar”))	39	9
(TITLE-ABS-KEY ({targeted proteomics}) AND TITLE-ABS-KEY ({Q-TOF}) AND NOT TITLE-ABS-KEY ({non-targeted proteomics}) AND NOT TITLE-ABS-KEY ({untargeted proteomics})) AND (LIMIT-TO (DOCTYPE, “ar”))	2	0
(TITLE-ABS-KEY ({targeted proteomics}) AND TITLE-ABS-KEY ({immunoassay}) AND NOT TITLE-ABS-KEY ({non-targeted proteomics}) AND NOT TITLE-ABS-KEY ({untargeted proteomics})) AND (LIMIT-TO (DOCTYPE, “ar”))	24	14

**Table 8:** Advantages and disadvantages of immunoassays and MS-based assays.

	Advantages	Disadvantages
Immunoassays	Low limit of detection (LOD) Low equipment cost Simplicity of implementation Unspecialized staff (low staff cost) High throughput rate	High reagents’ costs Ethical issues (animal use in antibody production) Specificity problems One component assay Kit–level variations
Mass Spectrometry	Highly specific Low limit of detection (LOD) Multi-component (multiplexing) analysis Low reagents’ costs	Challenging method development and implementation Specialized staff (high staff cost) High equipment cost (initial investment) Labor sample pre-treatment step Low throughput rate

## Data Availability

No new data were created or analyzed in this study.

## Supplementary Materials

The following supporting information can be downloaded at: https://www.mdpi.com/article/10.3390/molecules29235808/s1, Table S1: Specification of some modern mass analysers.
