# Supplementary material for: Current Role and Potential of Triple Quadrupole Mass Spectrometry in Biomedical Research and Clinical Applications
Source: Molecules. 2024 Dec 9;29(23):5808. doi: 10.3390/molecules29235808 (PMC11643727; doi:10.3390/molecules29235808)
Supplement: Supplementary file 1 [file molecules-29-05808-s001.zip › molecules-3217407-supplementary.pdf]

Table S1. Specification of some modern mass analysers *.

Vendor	MS model	Year of introduction	Collision Cell Clearance Time	IDL Sensitivity (positive/negative)	MRM Speed transitions/s	Mass Range, m/z	Minimum MRM Dwell Time, ms	Polarity Switch Speed, ms	Quad Resolution (FWHM), Da	Scan Speed, Da/s	Information source
Agilent	6475	2022	<1 ms	< 3.5 fg Reserpine / < 4 fg Chloramphenicol	500	5-3000	0.5	<25	0.7	18700	https://www.agilent.com/en/product/liquid-chromatography-mass-spectrometry-lc-ms/lc-ms-instruments/triple-quadrupole-lc-ms/6475-triple-quadrupole-lc-ms#specifications
	6495D	2023	<1 ms	n/d	700	5-3000	0.5	<25	0.7	18700	https://www.agilent.com/en/product/liquid-chromatography-mass-spectrometry-lc-ms/lc-ms-instruments/triple-quadrupole-lc-ms/6495-triple-quadrupole-lc-ms#specifications
		2017	<1 ms	<20 fg Reserpine on-column	500	5-1400	1	<20	0.7	15000	https://www.agilent.com/en/product/liquid-chromatography-mass-spectrometry-lc-ms/lc-ms-instruments/triple-quadrupole-lc-ms/ultivo-triple-quadrupole-lc-ms-lc-tq#specifications
Schimadzu	MS-8060NX	2020	n/d	n/d	Max. 555	2-2000	n/d	5	0.7	Max. 30,000	https://www.shimadzu.com/an/products/liquid-chromatograph-mass-spectrometry/triple-quadrupole-lc-msms/lcms-8060nx/spec.html
	MS-8060	2015	n/d	n/d	Max. 555	2-2000	n/d	5	0.7	Max. 30,000	https://www.shimadzu.com/an/products/liquid-chromatograph-mass-spectrometry/triple-quadrupole-lc-msms/lcms-8060/spec.html
	MS-8050	2013	n/d	n/d	Max. 555	2-2000	n/d	5	0.7	Max. 30,000	https://www.ssi.shimadzu.com/products/liquid-chromatograph-mass-spectrometry/triple-quadrupole-lc-msms/lcms-8050/spec.html
Schimadzu	MS-8045	2016	n/d	n/d	Max. 555	2-2000	n/d	5	0.7	Max. 30,000	https://www.shimadzu.com/an/products/liquid-chromatograph-mass-spectrometry/triple-quadrupole-lc-msms/lcms-8045/spec.html

Table S1. Specification of some modern mass analysers *.

Vendor	MS model	Year of introduction	Collision Cell Clearance Time	IDL Sensitivity (positive/negative)	MRM Speed transitions/s	Mass Range, m/z	Minimum MRM Dwell Time, ms	Polarity Switch Speed, ms	Quad Resolution (FWHM), Da	Scan Speed, Da/s	Information source
AB SCIEX	Triple Quad 3500	2014	n/d	n/d	n/d	5-2000	n/d	n/d	n/d		https://sciex.com/products/mass-spectrometers/triple-quad-systems/triple-quad-3500-system
	Triple Quad 4500	2012	n/d	n/d	n/d	5-2000	n/d	50	n/d	n/d	https://sciex.com/products/mass-spectrometers/triple-quad-systems/triple-quad-4500-system
	Triple Quad 5500+	2019	n/d	n/d	500	5-1250	n/d	5	n/d	12000	https://sciex.com/products/mass-spectrometers/triple-quad-systems/triple-quad-5500-lc-ms-ms-system-qtrap-ready
	Triple Quad 6500+	2016	n/d	n/d	n/d	2-2000	n/d	5 msec	n/d	12000	https://sciex.com/products/mass-spectrometers/triple-quad-systems/triple-quad-6500plus-system
AB SCIEX	Triple Quad 7500	2020	n/d	n/d	n/d	n/d	n/d	n/d	n/d	n/d	https://sciex.com/products/mass-spectrometers/triple-quad-systems/triple-quad-7500-system
Thermo Fisher Scientific	TSQ Quantis	2017	n/d	n/d	n/d	5-3000	n/d	n/d	n/d	n/d	https://www.thermofisher.com/order/catalog/product/TSQ02-10001?SID=srch-srp-TSQ02-10001
	TSQ Quantis Plus	2021	n/d	n/d	n/d	2-3000	n/d	n/d	n/d	n/d	https://www.thermofisher.com/order/catalog/product/TSQ03-11001?SID=srch-srp-TSQ03-11001

Table S1. Specification of some modern mass analysers *.

Vendor	MS model	Year of introduction	Collision Cell Clearance Time	IDL Sensitivity (positive/negative)	MRM Speed transitions/s	Mass Range, m/z	Minimum MRM Dwell Time, ms	Polarity Switch Speed, ms	Quad Resolution (FWHM), Da	Scan Speed, Da/s	Information source
Thermo Fisher Scientific	TSQ Altis	2017	n/d	n/d	n/d	5-2000	n/d	n/d	0.2	n/d	https://www.thermofisher.com/order/catalog/product/TSQ03-10002?SID=srch-srp-TSQ03-10002
	TSQ Altis Plus	2021	n/d	n/d	n/d	2-2010	n/d	n/d	n/d	n/d	https://www.thermofisher.com/order/catalog/product/TSQ03-10002?SID=srch-srp-TSQ03-10002
	TSQ Fortis	2017	n/d	n/d	n/d	2-3000	n/d	n/d	n/d	n/d	https://www.thermofisher.com/order/catalog/product/TSQ03-11003?SID=srch-srp-TSQ03-11003
	TSQ Fortis Plus	2021	n/d	n/d	n/d	2-3000	n/d	n/d	n/d	n/d	https://www.thermofisher.com/order/catalog/product/TSQ03-11003?SID=srch-srp-TSQ03-11003
Waters	Xevo TQ Absolute	2022	n/d	n/d	n/d	2-2048	n/d	n/d	n/d	n/d	https://www.waters.com/nextgen/us/en/products/mass-spectrometry/mass-spectrometry-systems/xevo-tq-absolute.html
	Xevo TQ-XS	2016	n/d	n/d	n/d	2-2048	n/d	n/d	n/d	n/d	https://www.waters.com/waters/en_US/Xevo-TQ-XS-Triple-Quadrupole-Mass-Spectrometry/nav.htm?locale=en_US&cid=134889751
	Xevo TQ-S	2010	n/d	n/d	n/d	2-2048	n/d	n/d	n/d	n/d	https://www.waters.com/waters/en_US/Xevo-TQ-S/nav.htm?locale=en_US&cid=10160596

Table S1. Specification of some modern mass analysers *.

Vendor	MS model	Year of introduction	Collision Cell Clearance Time	IDL Sensitivity (positive/negative)	MRM Speed transitions/s	Mass Range, m/z	Minimum MRM Dwell Time, ms	Polarity Switch Speed, ms	Quad Resolution (FWHM), Da	Scan Speed, Da/s	Information source
Waters	Xevo TQ-S micro	2014	n/d	n/d	n/d	2-2048	n/d	n/d	n/d	n/d	https://www.waters.com/waters/en_US/Xevo-TQ-S-micro-Triple-Quadrupole-Mass-Spectrometry/nav.htm?locale=en_US&cid=134798856
	Xevo TQ-S cronos	2019	n/d	n/d	n/d	2-2048	n/d	n/d	n/d	n/d	https://www.waters.com/waters/en_US/Xevo-TQ-S-cronos-Triple-Quadrupole-Mass-Spectrometry/nav.htm?locale=en_US&cid=135027354
	Xevo TQD	2011	n/d	n/d	n/d	2-2048	n/d	n/d	n/d	n/d	https://www.waters.com/webassets/cms/library/docs/6800_ic_xevo_tqd_capabilities_3-13.pdf
Bruker	EVOQ DART-TQ+	2023	n/d	n/d	1000	n/d	n/d	n/d	n/d	30000	https://www.bruker.com/en/products-and-solutions/mass-spectrometry/triple-quads/evoq-dart-tq-plus.html
	EVOQ LC-TQ	2012	n/d	n/d	n/d	n/d	n/d	n/d	n/d	n/d	https://www.bruker.com/en/products-and-solutions/mass-spectrometry/triple-quads/evoq-lc.html

* - information is taken from the official websites of the manufacturers listed in the table. It should be noted that the same model may have different values in specifications of different years; n/d – no data available, not all specification details are available on the manufacturers' sites. (accessed on for all links: 3 September 2024).
